# Comparison between Ischemic and Hemorrhagic Strokes in Functional Outcome at Discharge from an Intensive Rehabilitation Hospital

**DOI:** 10.3390/diagnostics11010038

**Published:** 2020-12-28

**Authors:** Emilia Salvadori, Gioele Papi, Greta Insalata, Valentina Rinnoci, Ida Donnini, Monica Martini, Catuscia Falsini, Bahia Hakiki, Annamaria Romoli, Carmen Barbato, Paola Polcaro, Francesca Casamorata, Claudio Macchi, Francesca Cecchi, Anna Poggesi

**Affiliations:** 1 IRCCS Fondazione Don Carlo Gnocchi, 50143 Florence, Italy; emilia.salvadori@unifi.it (E.S.); vrinnoci@dongnocchi.it (V.R.); idonnini@dongnocchi.it (I.D.); mmartini@dongnocchi.it (M.M.); cfalsini@dongnocchi.it (C.F.); bhakiki@dongnocchi.it (B.H.); annamariaromoli@gmail.com (A.R.); carmenbarbato88@gmail.com (C.B.); ppolcaro@dongnocchi.it (P.P.); fcasamorata@dongnocchi.it (F.C.); cmacchi@dongnocchi.it (C.M.); fcecchi@dongnocchi.it (F.C.); 2 NEUROFARBA Department, Neuroscience Section, University of Florence, 50134 Florence, Italy; iamgp22@gmail.com (G.P.); gretainsa@gmail.com (G.I.); 3 Department of Clinical and Experimental Medicine, University of Florence, 50134 Florence, Italy

**Keywords:** stroke, ischemic stroke, hemorrhagic stroke, rehabilitation, functional recovery

## Abstract

Comparison studies on recovery outcomes in ischemic (IS) and hemorrhagic strokes (HS) have yielded mixed results. In this retrospective observational study of consecutive IS and HS patients, we aimed at evaluating functional outcomes at discharge from an intensive rehabilitation hospital, comparing IS vs. HS, analyzing possible predictors. Modified Rankin Scale (mRS) at discharge was the main outcome. Out of the 229 patients included (mean age 72.9 ± 13.9 years, 48% males), 81 had HS (35%). Compared with IS (*n* = 148), HS patients were significantly younger (75 ± 12.5 vs. 68.8 ± 15.4 years, *p* = 0.002), required longer hospitalizations both in acute (23.9 ± 36.7 vs. 35.2 ± 29.9 days, *p* = 0.019) and rehabilitation hospitals (41.5 ± 31.8 vs. 77.2 ± 51.6 days, *p* = 0.001), and had more severe initial clinical deficit (mean number of neurological impairments: 2.0 ± 1.1 vs. 2.6 ± 1.4, *p* = 0.001) and mRS scores at admission (*p* = 0.046). At discharge, functional status change, expressed as mRS, was not significantly different between IS and HS (*F* = 0.01, *p* = 0.902), nor was the discharge destination (*p* = 0.428). Age and clinical severity were predictors of functional outcome in both stroke types. On admission in an intensive rehabilitation hospital, HS patients presented a worse functional and clinical status compared to IS. Despite this initial gap, the two stroke types showed an overlapped trajectory of functional recovery, with age and initial stroke severity as the main prognostic factors.

## 1. Introduction

Stroke is one of the world’s leading causes of death and disability. Ischemic stroke is nowadays considered a time-dependent disease due to the availability of acute treatments and represents 87% of all strokes [1]. Hemorrhagic strokes include intracerebral hemorrhage, representing 10% of all strokes, and aneurysmal subarachnoid hemorrhage, which represents 3% of all strokes [1]. In 2017, a total of 2.7 million individuals died of ischemic stroke, 3 million of intracerebral hemorrhage, and 0.4 million of aneurysmal subarachnoid hemorrhage [1]. Overall, the general prognosis of ischemic stroke is considered better than that of hemorrhagic stroke, in which death occurs especially in the acute and subacute phases [2,3].

Neurologic rehabilitation has the potential to affect functional outcomes in stroke patients by means of many different mechanisms [4]. Post-stroke recovery has been widely studied mainly in ischemic stroke, but as pathophysiology between ischemic and hemorrhagic forms is different, it could be hypothesized that also mechanisms of recovery and outcomes are dissimilar [5].

Comparisons between recovery outcomes in patients with ischemic and hemorrhagic stroke have yielded mixed results: Some studies, mainly community- or acute hospital-based, have found comparable activity limitation and recovery [5,6,7,8,9], whereas others have found greater recovery after hemorrhagic stroke [10,11,12,13]. Evidence of a similar or worse functional outcome in hemorrhagic stroke patients compared to ischemic ones mainly comes from community- or acute hospital-based studies, while better outcomes in hemorrhagic stroke patients were most common in studies conducted in the rehabilitation setting. Despite the few studies specifically focused on hemorrhagic stroke, available data show that the majority of motor recovery after hemorrhagic stroke seems to occur early, within the first 3–6 months [14,15,16].

More data are needed to better elucidate the recovery after rehabilitation in hemorrhagic strokes.

The present study is based on a retrospective revision of clinical records of stroke patients who underwent in-hospital intensive neurologic rehabilitation, and it is aimed at: (1) Evaluating and comparing functional outcomes of ischemic and hemorrhagic stroke patients at discharge from the intensive rehabilitation hospital; and (2) evaluating the predictive value of a large set of baseline characteristics, and of their possible interaction, on functional outcomes.

## 2. Materials and Methods

The present study was a single-center, retrospective observational study based on data collected from the revision of clinical records of consecutive stroke patients hospitalized for an intensive neurologic rehabilitation program. All adult (age > 18 years) patients discharged from the IRCCS Fondazione Don Carlo Gnocchi in Florence, Italy, with a diagnosis of stroke (ischemic or hemorrhagic) from 1 January 2018 to 30 June 2019 were included in the study. IRCCS Fondazione Don Carlo Gnocchi was a rehabilitation hospital with one highly specialized neurological intensive rehabilitation ward, i.e., severe acquired brain injury, mainly dedicated to patients with disorders of consciousness, and one intensive neurological rehabilitation ward. The individual rehabilitation project was developed and carried according to patient-centered objectives by an interdisciplinary team of health professionals. The study was conducted in accordance with the Helsinki Declaration and was approved by the local ethics committee.

The following data were collected from clinical records: (1) Socio-demographic characteristics (age, sex), vascular risk factors, and history of previous stroke; (2) length of stay (LOS) in the acute care hospital and the rehabilitation hospital; (3) classification of stroke type (ischemic vs. hemorrhagic) and subtypes (TOAST for ischemic strokes, and intracerebral vs. subarachnoid for hemorrhagic strokes); (4) types and overall burden of clinical deficits (motor deficits, aphasia, neglect, and dysphagia); (5) discharge destinations (home, other hospital, death).

### 2.1. Study Outcome

The primary outcome was the degree of dependence in daily activities as measured by means of the modified Rankin Scale (mRS) at discharge from the rehabilitation hospital [17]. The mRS was a broadly used disability scale ranging from 0 (no symptoms) to 6 (death) and the most widely used outcome measure in stroke clinical trials. As a primary outcome, in the present study, the mRS was used with two different approaches: (1) Distributions of mRS score at different time points, i.e., on admission and discharge, (2) change in mRS scores from admission to discharge.

### 2.2. Statistical Analyses

Descriptive analyses (means and standard deviations or frequencies and percentages) were used to illustrate the total sample characteristics. Independent sample *t*-tests and chi-squared tests were used to compare demographics, vascular risk factors, length of hospitalization and rehabilitation stay, and clinical characteristics between ischemic and hemorrhagic strokes. The presence of motor deficits (absent = 0, paresis = 1, plegia = 2), aphasia (absent = 0, present = 1), dysphagia (absent = 0, present = 1), and neglect (absent = 0, present = 1) were summed into a variable representing the total number of clinical deficits (range 0–5). The presence of the above mentioned neurological symptoms was defined based on the need of rehabilitation for the specific deficit.

For statistical analysis purposes, the mRS was analyzed according to different approaches. For the analyses of the distributions of mRS scores at different time points, non-parametric methods (Independent samples Mann–Whitney U and Jonckheere–Terpstra tests) were used for the comparisons between ischemic and hemorrhagic strokes. For the analyses of change in mRS scores during the stay in the rehabilitation setting, delta scores (Δs) were calculated by computing the difference between mRS scores obtained at admission and at discharge for each patient (positive score = improvement), and dichotomized as ‘not-improved’ (Δs ≤ 0) vs. ‘improved’ (Δs ≥ 1). The dichotomized functional outcome was then used to compare the rates of ischemic or hemorrhagic stroke patients with improvement, stratified according to mRS at admission (Mantel–Haenszel test). The mRS Δs were further used to calculate the efficiency and effectiveness scores according to the approach described by Paolucci et al. [11]. The efficiency score was computed by the following formula: mRS Δs/rehabilitation LOS, representing the average increase per day; the effectiveness score was computed by the following formula: mRS Δs/(initial score-maximum score)*100, representing the proportion of improvement achieved during rehabilitation. Independent samples *t*-tests were used to compare the efficiency and effectiveness scores between ischemic and hemorrhagic stroke patients.

Repeated measures ANOVA models were used to study the trajectory of mRS variations from admission to discharge in ischemic and hemorrhagic stroke patients (lesion type*time), taking into account the effect of sex, age, and total number of clinical deficits.

Logistic regression models on the association between baseline characteristics (demographics, vascular risk factors, and clinical deficits) used as potential predictors and the improvement in functional status (mRS Δs ≥ 1) used as the dependent variable, were carried out separately for ischemic and hemorrhagic strokes. Regression analyses were conducted in two steps: (1) Univariate models including each potential predictor independently (Models 1); (2) multivariate models including all predictors that resulted statistically significant in Models 1 (Models 2).

Within hemorrhagic stroke patients, the above-mentioned approaches were used to compare age and mRS scores between intracerebral hemorrhage and subarachnoid hemorrhage patients.

## 3. Results

From 1 January 2018 to 30 June 2019, 229 consecutive patients were discharged from the IRCCS Fondazione Don Carlo Gnocchi in Florence, Italy, with a diagnosis of stroke. Demographics and vascular risk factors are reported in Table 1: Mean (±SD) age was 72.9 ± 13.9 years and 48% (*n* = 111) were males. Considering stroke types, 148 (65%) patients had an ischemic stroke while 81 (35%) had a hemorrhagic stroke. The distribution of the subtypes of ischemic stroke according to the TOAST classification was as follows: 24 (16%) large-artery atherosclerosis, 56 (38%) cardioembolism, 16 (11%) small-vessel occlusion, 17 (11%) other determined etiology, and 35 (24%) undetermined etiology. Acute stroke treatments, i.e., thrombolysis and/or mechanical thrombectomy, were performed in 29 ischemic stroke patients (20%). Among the 81 patients with hemorrhagic stroke, 64 (79%) had an intracerebral hemorrhage, and 17 (21%) subarachnoid hemorrhage. Surgical interventions for hemorrhagic stroke were performed in 35 patients (43%) and included hematoma evacuation in 18 (28%) patients with intracerebral hemorrhage. All patients with subarachnoid hemorrhage underwent clipping or coiling procedures for the ruptured aneurysm.

Comparisons between stroke type in terms of demographics, vascular risk factors, and hospitalization characteristics showed that patients with hemorrhagic strokes were significantly younger, had less frequently atrial fibrillation, and stayed in the acute hospital for a longer period than patients with ischemic strokes (Table 1).

**Figure 1:**
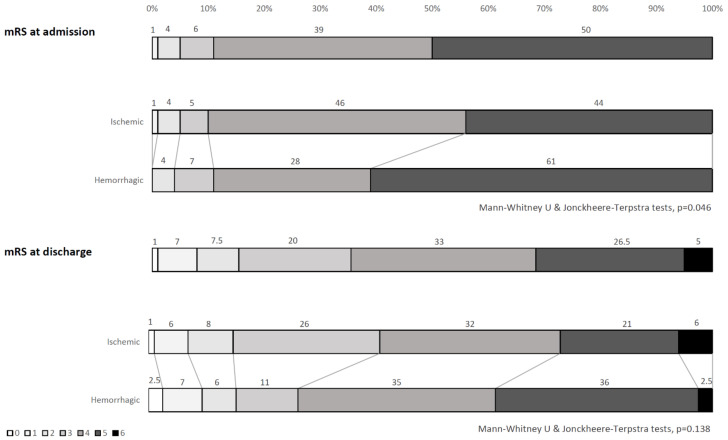
mRS score distributions at admission and discharge: Overall sample and comparisons between ischemic and hemorrhagic stroke patients.

As shown in Table 2, taking into account the clinical status at admission, patients with hemorrhagic strokes were more frequently admitted at the severe acquired brain injury ward, presented a higher burden of clinical deficits (particularly motor deficits and neglect), more often needed assisted nutrition, and remained in the rehabilitation setting for a longer time than patients with ischemic strokes. On admission, the mRS score distributions resulted as significantly different between ischemic and hemorrhagic stroke patients, with significantly higher disability among hemorrhagic patients (Figure 1).

**Figure 2:**
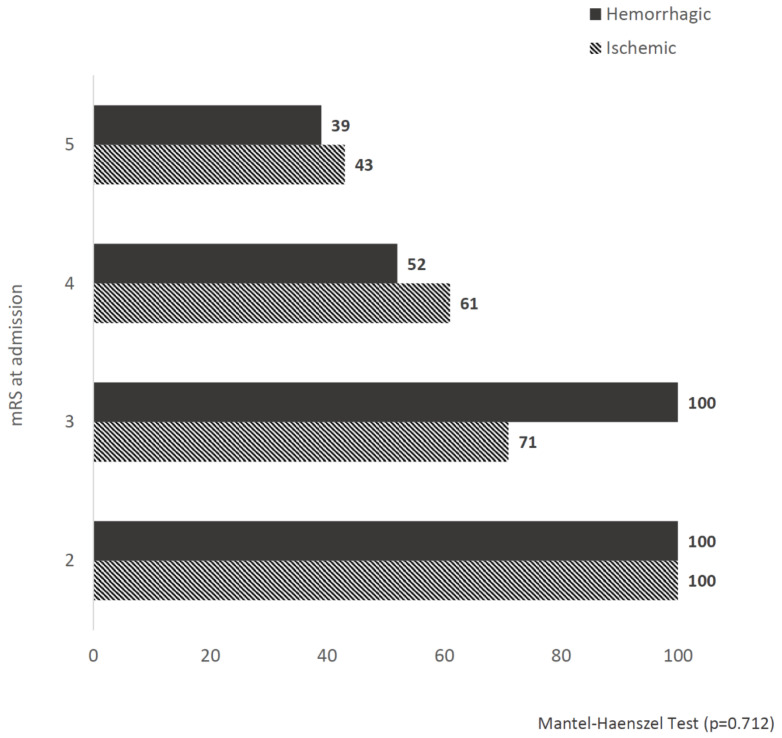
Rates of patients with an improved functional status (mRS Δs ≥ 1) at discharge stratified according to the mRS score distributions at admission: Comparisons between ischemic and hemorrhagic strokes.

At discharge, the mRS score distributions were not significantly different between ischemic and hemorrhagic strokes (Figure 1). After rehabilitation, 118O (52%) patients improved their mRS score after rehabilitation (mRS Δs ≥ 1). Figure 2 shows no statistically significant differences in the rates of patients with an improved functional status at discharge (mRS Δs ≥ 1) among ischemic or hemorrhagic stroke patients stratified according to the mRS score distributions at admission. No statistically significant differences between ischemic and hemorrhagic stroke patients resulted in the effectiveness (15.1 ± 23.5 vs. 16.4 ± 24.3, respectively, *p* = 0.698) and efficiency (0.012 ± 0.09 vs. 0.017 ± 0.03, respectively, *p* = 0.706) scores.

**Figure 3:**
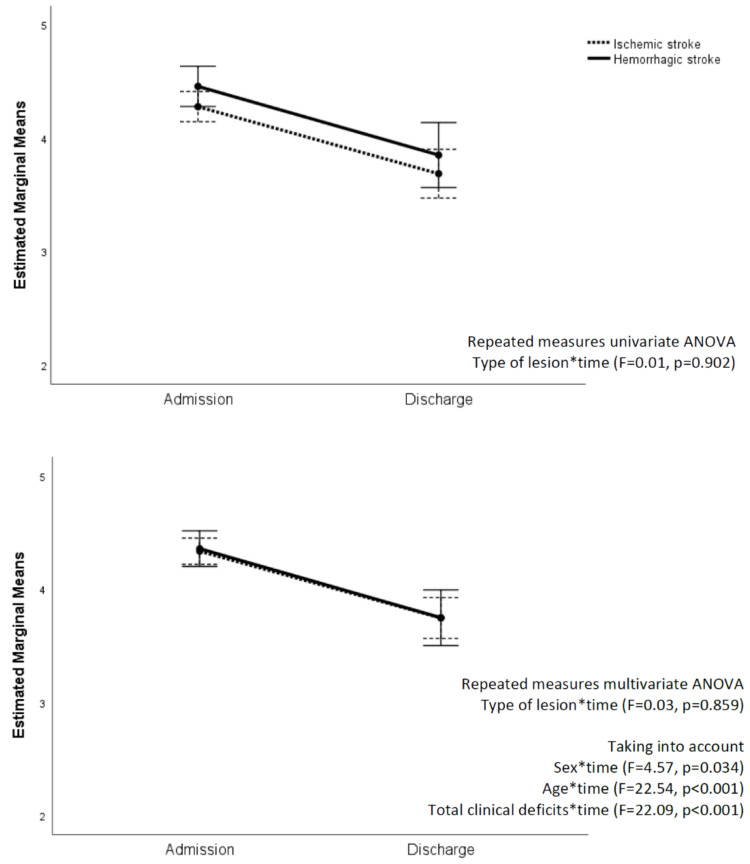
Variations in mRS scores from admission to discharge: Comparisons between ischemic and hemorrhagic strokes.

Taking into account the trajectory of the mRS scores from admission to discharge in ischemic and hemorrhagic stroke patients, the repeated measures univariate ANOVA showed no interaction between stroke type and time (*F* = 0.01, *p* = 0.902), and the overlap between the trajectories became even more evident taking into account the significant effects of sex (*F* = 4.57, *p* = 0.034), age (*F* = 22.54, *p* < 0.001), and total clinical deficits (*F* = 22.09, *p* < 0.001) (Figure 3). The same results were confirmed, also taking into account the effects of atrial fibrillation and LOS in an acute hospital as other possible influencing factors.

Looking at discharge destination, no differences emerged between stroke types. As for ischemic stroke patients, 56% returned home compared to 61% of the hemorrhagic ones, 37% were transferred to another hospital compared to 38% of the hemorrhagic strokes, and 6% died compared to 2% of hemorrhagic strokes (*p* = 0.428).

Multivariate logistic regression models on the association between baseline characteristics and functional outcome showed that age and overall burden of clinical deficits were significant predictors of functional improvement in both stroke types independently of sex, dyslipidemia, LOS in acute and rehabilitation hospitals, and mRS score at admission (Table 3). In the ischemic stroke group, female gender and shorter LOS in the acute hospital were also associated with a better outcome.

**Figure 4:**
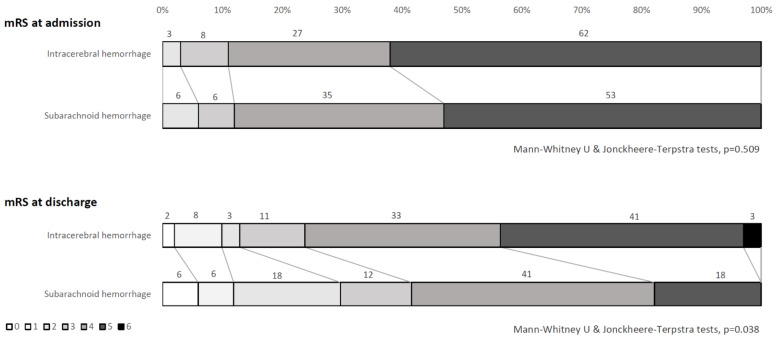
mRS score distributions at admission and discharge: Comparisons between intracerebral and subarachnoid hemorrhage patients.

Within hemorrhagic strokes, the comparison between intracerebral hemorrhage and subarachnoid hemorrhage showed no significant difference in age (70 ± 15.3 vs. 64.3 ± 15.6 years, respectively, *p* = 0.180) and in mRS score distributions at admission (Mann-Whitney U & Jonckheere-Terpstra tests, *p* = 0.509). A statistically significant difference resulted in mRS score distributions at discharge (Mann–Whitney U & Jonckheere–Terpstra tests, *p* = 0.038) (Figure 4), as well as in the rates of patients who improved (mRS Δs ≥ 1: 42% in intracerebral hemorrhage vs. 76% in subarachnoid hemorrhage, *p* = 0.012), and in the effectiveness (13.2 ± 22.6 vs. 28.1 ± 27.3, respectively, *p* = 0.047) and efficiency (0.013 ± 0.03 vs. 0.030 ± 0.04, respectively, *p* = 0.024) scores. All comparisons showed a better functional recovery in subarachnoid hemorrhage compared to intracerebral hemorrhage.

## 4. Discussion

The results of this study showed that hemorrhagic strokes presented a worse functional and clinical status compared to ischemic strokes at admittance in an intensive rehabilitation unit. Consistent with other studies, hemorrhagic stroke patients were younger, needed a longer and more intensive hospitalization both in acute and in rehabilitation settings, and suffered from more severe initial stroke severity than ischemic stroke ones [9,10,12].

Despite this initial gap, the two types of stroke showed an overlapped trajectory of functional recovery, also considering the effect of influencing factors such as age, sex, duration of stay in the rehabilitation hospital, and functional and clinical burden on admission. Results on the efficiency and effectiveness scores confirmed that, although hemorrhagic stroke patients started with a worse functional status and stayed longer in rehabilitation, the proportions of daily and overall improvement were the same in ischemic stroke patients.

Our results are not consistent with previous studies conducted in the rehabilitation setting that have found a greater recovery after hemorrhagic strokes [10,11,12,13]. Previous studies mainly excluded the more clinically complex or unstable stroke patients (e.g., subarachnoid hemorrhages, patients subjected to cerebral surgical interventions, or requiring acute hospital transfer for evaluation of medical complications), while our studies included all stroke patients consecutively admitted to the rehabilitation hospital without any clinical exclusion criteria. In line with this, in our sample, more than one-third of patients presented a hemorrhagic stroke, and this rate was higher than data from other studies aimed at the prognostic comparison with ischemic stroke. The availability of a severe acquired brain injury ward in the IRCCS Fondazione Don Carlo Gnocchi, allowed the inclusion in the study of a large sample of hemorrhagic stroke patients, representative also of the more clinically complex ones.

Among demographics, vascular risk factors, and clinical characteristics taken into consideration as possible predictors of improvement, age, and initial stroke severity were confirmed as the main prognostic factors both in ischemic and hemorrhagic stroke patients. Our results are in line with the consistent findings across studies about the relevance of these prognostic factors in both stroke types [10,12,13,14]. Interestingly, women seemed to have a better outcome in the ischemic stroke group. Increasing evidence suggests the existence of gender differences in stroke etiology, clinical presentation, treatment, and outcome. Preliminary data point toward a poorer post-stroke outcome for women than for men, but further studies are need to elucidate the effect of a variety of potential confounding factors [18,19]. The relation between functional outcome and gender will be the focus of another publication derived from the same study (paper submitted).

The present study has limitations that need to be considered. The retrospective design of the study influenced the type of variables to be collected, as well as their completeness and accuracy, and thus the validity of the results. Some relevant clinical variables, such as comorbidities, cardioembolic causes, and medications, were not systematically available for all patients, and we decided not to include partial data in our analyses. In line with this, the choice of the mRS as the main functional outcome was driven by the availability of this score for all patients at admittance and at discharge. Even though mRS is recognized as a valid instrument for clinical trials in acute stroke, its reliability and sensitivity to change in a rehabilitation setting may be limited by the use of large categories, whose scoring is based on the overall impression of function, and by the clinical significance of any change between the categories [17]. Furthermore, the overall clinical status was estimated based on the presence of the most relevant neurological deficits because no clinical scale validated for the assessment of the neurological status following a stroke, e.g., the NIH Stroke Scale (NIHSS), was available for all patients and could be used in the analyses.

Our findings may not be generalized to all patients with stroke but are representative only of moderate-to-severe stroke patients who undergo intensive neurologic rehabilitation. On the one hand, the inclusion of all consecutive stroke patients without any exclusion criterion based on clinical severity gave us the opportunity to study the outcome of a ‘real world’ sample of post-acute stroke patients undergoing intensive inpatient rehabilitation. On the other hand, the inclusion of clinically complex patients may have increased the impact of possible confounding factors that are difficult to manage within a retrospective study design.

Despite the above-mentioned methodological limitations, our study represents a contribution in the field of comparison between recovery outcomes after rehabilitation in patients with ischemic and hemorrhagic strokes and may be useful in guiding the development of future prospective studies. Functional recovery in hemorrhagic strokes has for many years been neglected due to its known negative prognosis compared to ischemic strokes. Despite the higher mortality rates during the first phases after a hemorrhagic stroke, the final functional recovery seems to happen and seems to be as much as effective as in ischemic strokes. More research efforts are needed to investigate the possible different mechanisms in neural repair among the different stroke types, with the final aim of delineating the best clinical care pathways and rehabilitation programs.

## Figures and Tables

**Table 1:** Demographic, vascular risk factors, and hospitalization stay of the study cohort, and comparisons between ischemic and hemorrhagic strokes.

Variables	Total Cohort	Ischemic Stroke	Hemorrhagic Stroke	*p*
	*n* = 229	*n* = 148	*n* = 81	
Age (years)	72.9 ± 13.9	75 ± 12.5	68.8 ± 15.4	0.002 *
Sex (male)	111 (48%)	74 (50%)	37 (46%)	0.532 ^#^
Hypertension	184 (80%)	118 (80%)	66 (81%)	0.750 ^#^
Diabetes	53 (23%)	39 (26%)	14 (17%)	0.120 ^#^
Dyslipidemia	50 (22%)	35 (24%)	15 (18%)	0.369 ^#^
Atrial fibrillation	66 (29%)	55 (37%)	11 (14%)	0.001 ^#^
Smoking habits	88 (38%)	54 (36%)	34 (42%)	0.414 ^#^
History of previous stroke	22 (10%)	13 (9%)	9 (11%)	0.568 ^#^
Pre-stroke mRS (score)	0.6 ± 1.1	0.7 ± 1.2	0.5 ± 0.9	0.362 °
Pre-Stroke mRS				
0	158 (69%)	92 (67%)	51 (72%)	0.362 ^#^
1	30 (13%)	19 (14%)	9 (13%)	
2	18 (8%)	8 (6%)	8 (11%)	
3	18 (8%)	13 (9%)	3 (4%)	
4	5 (2%)	5 (4%)	0 (0%)	
Referral hospital		68 (46%)	37 (46%)	0.969 ^#^
Careggi University Hospital	105 (46%)			
Other acute care hospital	124 (54%)			
LOS in acute hospital (days)	27.9 ± 34.8	23.9 ± 36.7	35.2 ± 29.9	0.019 *

LOS: Length of stay; * Independent samples *t* tests; ^#^ Chi square tests; ° Independent samples Mann–Whitney U test.

**Table 2:** Rehabilitation stay and clinical characteristics of the study cohort, and comparisons between ischemic and hemorrhagic strokes.

Variables	Total Cohort	Ischemic Stroke	Hemorrhagic Stroke	*p*
	*n* = 229	*n* = 148	*n* = 81	
Rehabilitation ward				
Severe acquired brain injury	59 (26%)	20 (13%)	39 (48%)	0.001 ^#^
Neurologic rehabilitation	170 (74%)	128 (87%)	42 (52%)	
LOS in rehabilitation hospital (days)	54.1 ± 43.4	41.5 ± 31.8	77.2 ± 51.6	0.001 *
Motor deficit				
Absent	23 (10%)	17 (12%)	6 (7.5%)	0.023 ^#^
Paresis	120 (52%)	85 (57%)	35 (43%)	
Plegia	86 (38%)	46 (31%)	40 (49.5%)	
Aphasia	78 (34%)	46 (31%)	32 (39%)	0.198 ^#^
Neglect	52 (23%)	26 (18%)	26 (32%)	0.012 ^#^
Dysphagia	88 (38%)	50 (34%)	38 (47%)	0.051 ^#^
Nutrition				
OS	142 (63%)	106 (72%)	36 (46%)	0.001 ^#^
Nasogastric tube	37 (16%)	22 (15%)	15 (19%)	
Percutaneous endoscopic gastrostomy	47 (21%)	19 (13%)	28 (35%)	
Total number of clinical deficits (range 0–5)	2.2 ± 1.3	2.0 ± 1.1	2.6 ± 1.4	0.001 *

LOS: Length of stay; * Independent samples *t* tests; ^#^ Chi square tests.

**Table 3:** Association between demographics, vascular risk factors, and clinical deficits and improvement in functional status (mRS Δs ≥ 1) at discharge.

Variables	Ischemic Stroke				Hemorrhagic Stroke			
	Model 1		Model 2		Model 1		Model 2	
	OR (95% CI)	*p*	OR (95% CI)	*p*	OR (95% CI)	*p*	OR (95% CI)	*p*
Age (years)	0.97 (0.94–0.99)	0.046	0.95 (0.91–0.98)	0.004	0.97 (0.95–1.00)	0.093	0.95 (0.91–0.99)	0.015
Sex (female)	1.97 (1.01–3.83)	0.046	2.51 (1.17–5.39)	0.018	1.06 (0.44–2.53)	0.904	1.62 (0.53–4.99)	0.397
Hypertension	0.95 (0.42–2.15)	0.903	-	-	0.82 (0.27–2.53)	0.735	-	-
Diabetes	1.13 (0.54–2.37)	0.742	-	-	2.09 (0.63–6.89)	0.226	-	-
Dyslipidemia	0.94 (0.43–2.03)	0.870	0.75 (0.32–1.78)	0.513	3.51 (1.01–12.16)	0.048	4.34 (0.99–18.96)	0.051
Atrial fibrillation	0.53 (0.27–1.06)	0.073	-	-	0.83 (0.23–2.98)	0.779	-	-
Smoking habits	0.93 (0.49–1.91)	0.931	-	-	0.69 (0.29–1.69)	0.421	-	-
History of previous stroke	0.58 (0.17–1.91)	0.369	-	-	1.32 (0.33–5.32)	0.695	-	-
LOS in acute hospital (days)	0.98 (0.97–0.99)	0.038	0.98 (0.96–0.99)	0.040	0.99 (0.97–1.01)	0.196	0.98 (0.96–1.01)	0.216
LOS in rehabilitation hospital (days)	0.99 (0.98–1.01)	0.398	0.99 (0.98–1.01)	0.655	0.99 (0.98–0.99)	0.041	1.00 (0.99–1.01)	0.985
mRS score (admission)	0.65 (0.42–1.01)	0.055	1.14 (0.64–2.03)	0.653	0.33 (0.1–0.69)	0.003	0.60 (0.23–1.60)	0.312
Total number of clinical deficits (range 0–5)	0.65 (0.4–0.88)	0.005	0.58 (0.39–0.85)	0.003	0.56 (0.39–0.79)	0.001	0.61 (0.38–0.97)	0.037

Model 1: Univariate logistic regression models, including each potential predictor independently; Model 2: Multivariate logistic regression model including all predictors that resulted statistically significant in Models 1.

## Data Availability

Data are available from the corresponding author upon reasonable request.
